# Oral mucosal melanoma treated with carbon ion radiotherapy: a case report

**DOI:** 10.1186/s13256-016-1071-8

**Published:** 2016-10-18

**Authors:** Atsushi Musha, Jun-ichi Saitoh, Katsuyuki Shirai, Satoshi Yokoo, Tatsuya Ohno, Takashi Nakano

**Affiliations:** 1 Gunma University Heavy Ion Medical Center, 3-39-22 Showa-machi, Maebashi, Gunma Japan; 2 Department of Stomatology and Maxillofacial Surgery, Gunma University Graduate School of Medicine, 3-39-22 Showa-machi, Maebashi, Gunma Japan

**Keywords:** Oral mucosal melanoma, Head and neck tumor, Carbon ion radiotherapy, Concomitant chemotherapy

## Abstract

**Background:**

Oral mucosal melanoma is a rare disease with a relatively poor prognosis. Carbon ion radiotherapy has been shown to be effective against radiotherapy-resistant tumors owing to its excellent dose concentration and high biological effect.

**Case presentation:**

Our patient was a 66-year-old Japanese man with oral mucosal melanoma of his right maxillary gingiva (T4aN0M0). He received carbon ion radiotherapy at 57.6 Gy (relative biological effectiveness) in 16 fractions for 4 weeks. Concomitant chemotherapy (dacarbazine + nimustine + vincristine) was administered at the same time as carbon ion radiotherapy initiation. Two courses of adjuvant chemotherapy were given after carbon ion radiotherapy. Although he experienced grade 2 acute oral mucositis, his symptoms improved within a few weeks of undergoing carbon ion radiotherapy. He was alive at the time of reporting, 35 months after treatment, without any recurrence. Late toxicity has not been observed.

**Conclusions:**

Carbon ion radiotherapy for oral mucosal melanoma resulted in a good local effect.

## Background

Oral mucosal melanoma (OMM) is a rare disease with a relatively poor prognosis. The 5-year overall survival rates as reported by a number of institutions in which the disease was treated with surgery, postoperative radiotherapy, and adjuvant chemotherapy ranged from 10 to 35 % [1–4]. Carbon ion radiotherapy (C-ion RT) has been shown to be effective against radiotherapy-resistant head and neck tumors owing to its excellent dose concentration and high biological effect [5]. As C-ion RT has excellent dose localization, the radiation dose to normal tissues is minimal. C-ion RT is reported to be effective for mucosal melanoma because C-ion beams show an increase in energy deposition as depth increases and they have stronger biological effects than X-rays [6]. The OMM usually receives a high dose of irradiation over a broad area of the target region including the neighboring mucosa, and chemotherapy may also be administered. Consequently, it is important to assess acute and late toxicities as well as tumor control. However, there have been no previous specific reports on the clinical course of OMM after C-ion RT. We present a case report 35 months after OMM was successfully treated with C-ion RT combined with chemotherapy.

## Case presentation

**Fig. 1:**
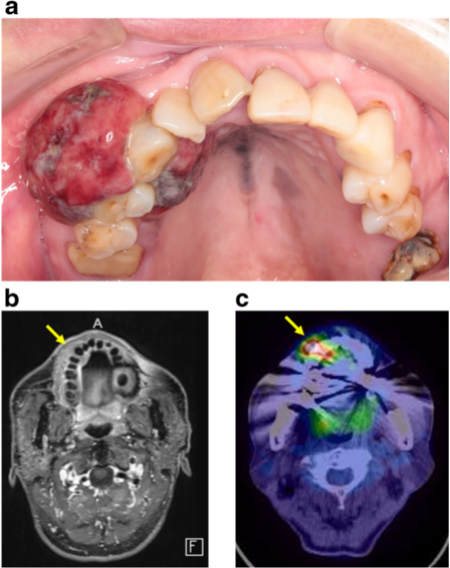
A 66-year-old man with mucosal melanoma of the right maxillary gingiva and palate. (**a**) Intraoral photograph before carbon ion radiotherapy (C-ion RT). (**b**) Gadolinium-enhanced T1-weighted magnetic resonance image before C-ion RT. Yellow arrow revealed a mass on the maxillary gingiva (16 × 10 mm). (**c**) F-18 fluorodeoxyglucose positron emission tomography image before C-ion RT. Yellow arrow revealed abnormal accumulation in the tumor

**Fig. 2:**
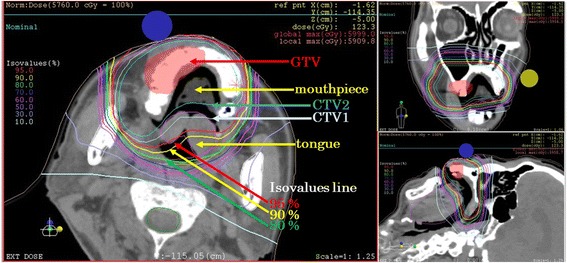
Dose distribution of carbon ion radiotherapy. Treatment plan for the oral mucosal melanoma. The gross tumor volume is shown in *red*. We used a shrinking field around clinical target volume 1 and clinical target volume 2. Clinical target volume 1 is shown by the *gray line* on the anterior plan. Clinical target volume 2 is shown by the *cyan line* on the posterior plan. An isovalues line of 95 % is shown by the *red line* and almost covers the clinical target volume. *CTV* clinical target volume, *GTV* gross tumor volume

A 66-year-old Japanese man presented to a general hospital, where mucosal melanoma of his right maxillary gingiva was confirmed on biopsy. The mass was present in his right maxillary gingiva, and a black lesion was present across a wide extent of his palate (Fig. 1a). At presentation, magnetic resonance imaging (MRI) revealed a mass of 16×10 mm on his maxillary gingiva (Fig. 1b). ^18^F-fluorodeoxyglucose positron emission tomography revealed abnormal accumulation in the tumor (Fig. 1c). He was diagnosed with T4aN0M0, stage IVA mucosal melanoma of his right maxillary gingiva. Surgery with a safe margin was possible; however, C-ion RT was selected based on postoperative functional and aesthetic considerations and our patient’s preference (Fig. 2). A total dose of 57.6 Gy (relative biological effectiveness; RBE) in 16 fractions was administered. Physical dose calculations were performed using the pencil beam algorithm. The clinical dose distribution was calculated according to the physical dose and the RBE. The dose of C-ion RT was expressed as “Gy (RBE)”: physical C-ion dose (Gy)×RBE. He was positioned in customized cradles (Moldcare, Alcare, Tokyo, Japan) and immobilized using a thermoplastic shell (Shellfitter, Kuraray, Osaka, Japan). A customized mouthpiece was used to fix the teeth of both his jaws and to maintain the position of his lower jaw. Computed tomography (CT) images with a 2-mm thickness were acquired for treatment planning, which used MRI as a reference. A margin of at least 5 mm was added to the gross tumor volume (GTV) to define the clinical target volume (CTV). CTV1 included the whole of each anatomical site (gum, palate, and maxillary sinus), while CTV2 was limited to the GTV and mucosal melanosis. Planning target volume (PTV) 1 and PTV2 had margins of 2 mm added around CTV1 and CTV2, respectively. PTV1 was irradiated initially with 32.4 Gy (RBE)/9 fractions, and thereafter; PTV2 was irradiated to a total dose of 57.6 Gy (RBE)/16 fractions. Organs at risk (OARs; the eye, optic nerve, optic chiasm, inner ear, brain stem, spinal cord, mandible, palate, and tongue) were outlined on the planning CT scan for treatment planning and dose-volume histogram analysis. Treatment planning was performed using a XiO-N system (Elekta AB, Stockholm, Sweden). The composite dose distribution is shown in Fig. 2.

**Fig. 3:**
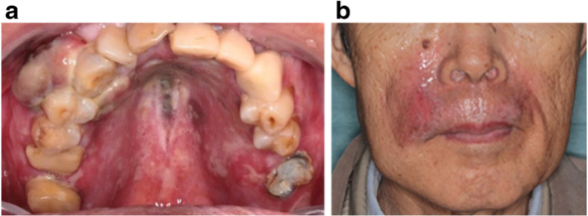
Acute radiation mucositis at the palate and acute radiation dermatitis were observed, both of which were classified as grade 2 based on the Common Terminology Criteria for Adverse Events, version 4.0. **a** Grade 2 acute radiation mucositis occurred shortly after carbon ion radiotherapy administration. **b** Grade 2 acute radiation dermatitis occurred 2 weeks after carbon ion radiotherapy administration

Acute radiation mucositis at his palate and acute radiation dermatitis were observed, both of which were classified as grade 2 based on the Common Terminology Criteria for Adverse Events (CTCAE), version 4.0 (Fig. 3). His mucositis and dermatitis resolved 1 month after C-ion RT treatment. Three-course concomitant chemotherapy (Day 1, 120 mg/m^2^ dacarbazine, 70 mg/m^2^ nimustine, and 0.7 mg/m^2^ vincristine; Day 2 to 5, 120 mg/m^2^ dacarbazine) with a 4-week interval was administered, with the first course administered at C-ion RT initiation, the second course at C-ion RT completion, and the third course 4 weeks after the second course.

**Fig. 4:**
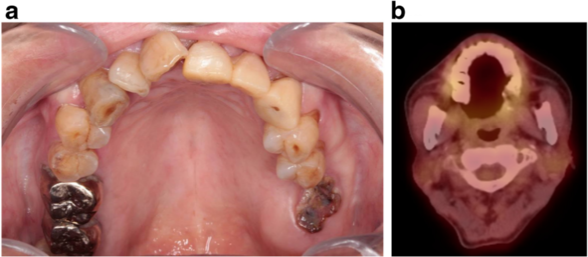
**a** Intraoral photograph 35 months after carbon ion radiotherapy. **b** ^18^F-fluorodeoxyglucose positron emission tomography image 35 months after carbon ion radiotherapy

Our patient did not experience any chronic adverse events, and a complete disease response was apparent 35 months after the C-ion RT without any signs of recurrence (Fig. 4). There were no other adverse events such as dysgeusia, xerostomia, radio-osteonecrosis, or the loss of a tooth.

## Discussion

Surgery along with chemotherapy remains the treatment of choice in mucosal melanoma of the head and neck [7]. However, large tumors or those involving adjacent critical structures often cannot be completely resected due to aesthetic considerations or functional reasons. Postoperative radiotherapy reduces the risk of locoregional recurrence in patients with head and neck mucosal melanoma, and locoregional recurrence was in turn shown to be an independent risk factor for overall survival in a systematic review [8]. C-ion RT allows a highly localized delivery of energy that can increase the radiation dose to the tumor while minimizing the irradiation of adjacent normal tissues. The present case showed good tumor control without late severe toxicity. If radical surgery was chosen for this case, it would have been difficult to conserve the teeth and gingiva adjacent to the tumor.

C-ion RT was first performed at the National Institute of Radiological Sciences in Japan. Initially, patients with malignant melanoma in the head and neck were treated with C-ion RT alone [6]. The overall survival rates at 3 and 5 years were 46.1 % and 27.0 %, respectively, which are similar to the most favorable results obtained using surgery with or without radiotherapy or chemotherapy [1–3]. This study strongly suggested the need for additional systemic therapy to prevent distant metastasis. Thereafter, the first course of concomitant chemotherapy with dacarbazine, nimustine, and vincristine (DAV) was administered at the same time as C-ion RT initiation, the second course upon C-ion RT completion, and the third course subsequently [5]. Although the local control rate remained almost unchanged, the 3-year survival rate improved from 46.1 % to 65.3 % due to the concomitant chemotherapy [9]. In contrast, the 3-year survival rate of head and neck melanomas (sinonasal) treated with proton therapy was 68.0 %, almost identical to that achieved using C-ion RT with DAV therapy. DAV therapy makes little contribution to the systemic effect. In the case we report here, the patient had survived for 35 months as a result of the good local effect achieved using C-ion RT. The 5-year survival rate with DAV therapy for general site melanoma was found to be 46.2 % in a historic study [10]. Head and neck malignant melanoma may be different from malignant melanoma of other sites with respect to its response to treatment. In a more recent study, it was established that immuno-checkpoint inhibitors, including anti-programmed cell death protein 1 (anti-PD-1) and anti-cytotoxic T-lymphocyte antigen-4 (anti-CTLA-4) antibodies, were important treatment options for advanced melanoma [11]. The objective-response rate was 40 %, and clinical activity was observed in 65 % of patients [11]. Therefore, immunotherapy may supersede DAV therapy, as immuno-checkpoint inhibitors have replaced chemotherapy. Two previous studies included two patients with C-ion RT alone [6], and seven with C-ion RT and concomitant therapy [9]; however, neither study reported subgroup analyses for the OMM in terms of efficacy and safety of C-ion RT with or without chemotherapy.

The prognosis of malignant melanoma is influenced by the presence of distant metastasis. Lymph node metastases are present in 25 % of patients with oral cavity melanoma, and the likelihood of lymph node metastases increases when the thickness of these lesions is >5 mm [1]. Consequently, when a patient has a tumor with a thickness of ≥5 mm, follow-up should be more rigorous. We perform neck dissection whenever isolated lymph node metastasis in the neck is detected. In our case, a good treatment response with C-ion RT was apparent 35 months after treatment. Based on previous reports of treatment for malignant melanoma in the head and neck, the 5-year local control rate is >80 % with C-ion RT [6, 10] and 62.0 % for proton therapy [12], and the administration of C-ion RT can thus potentially improve the quality of life of patients.

Fuji *et al*. reported delayed recurrence (47 months and 50 months) of malignant melanoma in the head and neck region after proton therapy [12]. Therefore, further follow-up will be necessary to confirm long-term efficacy.

## Conclusion

C-ion RT for OMM provided good local control after 35 months.

## Abbreviations

anti-CTLA-4: Anti-cytotoxic T-lymphocyte antigen-4
anti-PD-1: Anti-programmed cell death protein 1
C-ion RT: Carbon ion radiotherapy
CT: Computed tomography
CTCAE: Common Terminology Criteria for Adverse Events
CTV: Clinical target volume
DAV: Dacarbazine, nimustine, and vincristine
GTV: Gross tumor volume
MRI: Magnetic resonance imaging
OARs: Organs at risk
OMM: Oral mucosal melanoma
PTV: Planning target volume
RBE: Relative biological effectiveness
